# Integrating social and behavioral determinants of health into patient care and population health at Veterans Health Administration: a conceptual framework and an assessment of available individual and population level data sources and evidence-based measurements

**DOI:** 10.3934/publichealth.2019.3.209

**Published:** 2019-07-03

**Authors:** Elham Hatef, Zachary Predmore, Elyse C. Lasser, Hadi Kharrazi, Karin Nelson, Idamay Curtis, Stephan Fihn, Jonathan P. Weiner

**Affiliations:** 1 Center for Population Health IT, Department of Health Policy and Management, Johns Hopkins Bloomberg School of Public Health, Baltimore, MD, USA; 2 Veterans Affairs Puget Sound Health Care System, Seattle, WA, USA; 3 Department of Medicine, University of Washington School of Medicine, Seattle, WA, USA

**Keywords:** patient-centered medical home, patient care, population health, social and behavioral determinants of health, veterans health administration

## Abstract

The premise of this project was that social and behavioral determinants of health (SBDH) affect the use of healthcare services and outcomes for patients in an integrated healthcare system such as the Veterans Health Administration (VHA), and thus individual patient level socio-behavioral factors in addition to the neighborhood characteristics and geographically linked factors could add information beyond medical factors mostly considered in clinical decision making, patient care, and population health. To help VHA better address SBDH risk factors for the veterans it cares for within its primary care clinics, we proposed a conceptual and analytic framework, a set of evidence-based measures, and their data source. The framework and recommended SBDH metrics can provide a road map for other primary care-centric healthcare organizations wishing to use health analytic tools to better understand how SBDH affect health outcomes.

## 1. Introduction

Social and Behavioral Determinants of Health (SBDH), a combination of behavioral, social, economic, environmental, and occupational factors, are powerful drivers of morbidity, mortality, and future well-being, yet they mostly lie outside the domain of the conventional medical care delivery system [1]. While these modifiable behaviors and community exposures (defined either geographically or by other means such as race) play a significant role in 60% of preventable deaths in the U.S., direct medical services account for more than 95% of the trillion dollars spent on healthcare annually [2]. Despite U.S. having the highest per capita medical expenditures of any country in history, the limited investments in non-medical services might play a role in the U.S. health indicators lagging behind most other high-income nations [3].

To help mitigate potential negative impacts of key SBDH risk factors, healthcare organizations need to address these underlying factors at the overall neighborhood level as well as within focused sub-groups of high-risk individuals. Investment in behavioral and social services, and connecting high-need patients to these services, would improve the health outcomes and reduce the cost of services for healthcare organizations [4]. For instance, a reduction of preventable hospitalization among residents of low-income neighborhoods in the U.S. to the level of those living in high-income neighborhoods would lead to 500,000 fewer hospitalizations per year, saving $3.6 billion in hospitalization costs [4].

In the last several years, U.S. federal, state, and private payers have initiated a series of policies and programs to improve the value of healthcare. These initiatives generally emphasize financial incentives to increase the quality of care and reduce healthcare costs [5]. In order for clinicians and health delivery systems to navigate and succeed within these “pay for performance” programs, they must better address social and behavioral risk factors within their target population. Some of these factors are linked to the individual patient, but where the patient lives is also a major determinant, or at least a correlate, of SBDH related risk [6]–[8]. Without considering SBDH factors in decision-making and program development, the special needs of high-cost patients who are concomitantly facing socioeconomic challenges and behavioral health problems might not be properly addressed thus resulting in providers to be penalized financially [6],[7]–[9].

Therefore, understanding patients' individual social and behavioral risk factors in addition to the socio-environmental context of the neighborhood in which the patient resides and the impact of those socio-environmental inequalities on behavioral factors is critical to assess the level of achievement of health and wellbeing [7]. Accordingly, for healthcare organizations which provide services to patients with social and behavioral risk factors and those from the neighborhoods with significant levels of non-medical risk factors, a formal assessment of the SBDH related challenges and needs for individual patients and within patients' communities is essential for designing both individual and population level responses and interventions. Without this SBDH assessment it would be difficult, if not impossible, to provide good care or to succeed within value-based delivery systems [8],[9].

Despite the importance and significant impact of SBDH and place of living on health, medical care providers rarely have tools available to incorporate information about patients' social and behavioral risks, and neighborhood characteristics into clinical or organizational decision-making [1]. Inclusion of such individual-level SBDH as well as environmental SBDH related risks into decision-making frameworks requires a systematic approach to identify and respond to those factors that have the highest impact on health outcomes and cost [10],[11]. This response can occur either at the patient care level, for subpopulations (e.g., for a cohort enrolled within a patient-centered medical home or PCMH), or for a neighborhood at-large (e.g., certain target neighborhoods, city, county, or higher level) [12],[13].

As one of the largest integrated health systems in the country, the Veterans Health Administration (VHA) is increasingly interested in the development of tools and health indicators that take into account SBDH factors of the veterans and those associated with their communities [14]–[16]. Unlike most medical providers, the VHA has a statutory commitment to address both the medical and non-medical needs of their patients.

## 2. Development of the evidence-based framework and selection of social and behavioral risk measures

The overarching goal of the project reported here was to help VHA better address SBDH risk factors for the veterans it cares within its PCMH program. In the VHA, these primary care organizational units are termed Patient-Aligned Care Teams, or PACT [17]. The premise of this project was that these SBDH factors affect the use of healthcare services and outcomes for each of the VHA's primary care clinics, and thus individual patient level socio-behavioral factors in addition to the geographically linked factors could add information beyond medical factors mostly considered in clinical decision making, patient care, and population health. While some of these modifiable SBDH risk factors can be addressed at a primary care clinic, there are community exposures that must be addressed by population-level programs.

To achieve this goal we first undertook a literature review and environmental scan which guided us to develop a conceptual framework. The framework showed how practices could use such social, behavioral, or geo-linked data for patient care, risk prediction models, risk adjustment programs, or population-level analytics for population health. The review also resulted in proposing an initial evidence-based set of measures and available data sources. The measures, using recommended SBDH domains by the National Academy of Medicine [18] were reflective of individual and population-level SBDH relevant to primary care settings that could, for most patients and neighborhoods, be documented based on existing electronic data sets. We narrowed the list of SBDH domains after completing the literature review, consulting with clinicians and researchers who collected and used SBDH data regularly, gauging the basic data availability of domain-specific SBDH factors, and high-level VHA priorities.

While we did not perform a systematic literature review our review included a number of databases such as PubMed, Web of Science, and Google Scholar. We used the articles identified through the PubMed search to start a snowball sample. In addition, we selected gray literature found via Google. We used keywords such as “Patient-Centered Care”, “Social and Behavioral Determinants of Health”, and “Electronic Medical Records”. The search resulted in 1848 articles. After an initial assessment we narrowed down our review to selected articles that utilized social, behavioral, and geo-coded data in primary care settings, specifically in PCMH clinics. We included conceptual papers and commentaries, as well as case studies or quantitative analyses of a single site or program. We also included studies addressing the role of social and behavioral factors in assessment of hospitalization and readmission in a primary care setting.

## 3. Conceptual framework and integration of patient and population—level data

**Figure 1.**
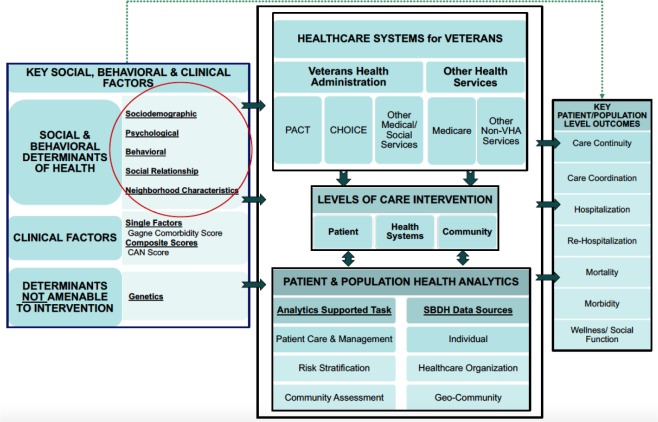
A conceptual framework for integrating patient and population-level data to address social and behavioral determinants of health in the VHA's primary care clinics.

Figure 1 presents an evidence-based framework to address SBDH and to integrate them in the analytic platforms in the VHA's PCMH program [1],[11],[19]–[21]. It presents the conceptual representation of the VHA's delivery system, including relevant inputs, throughputs, and outputs. Specifically, the graphic outlines *Key Social, behavioral, and Clinical Risk Factors*—in the leftmost box—as important inputs and categories of *Key Patient and Population Level Outcomes*—in the rightmost box—as key outputs of care provided to veterans enrolled in the VHA's PCMH program. We posit that ideally all of these factors need to be measured, monitored, and managed. Social and Behavioral risk factors are listed in details in table 1 and the appendix table. The tables also include a comprehensive list of evidence-based measures and their data sources.

CAN Score: Care Assessment Needs Score which reflects estimated probability of admission or death within a specified time period (90 days or 1 year); CHOICE Program: a benefit that allows eligible veterans to receive healthcare from a community provider rather than waiting for a VHA appointment or traveling to a VHA facility; PACT: Patient-Aligned Care Team, type of Patient-Centered Medical Home; SBDH: Social and Behavioral Determinants of Health; VHA: Veterans Health Administration.

The large middle box depicts the care delivery system for veterans. At the top we summarize the key structural components, including the PACT—the PCMH initiative, the CHOICE program—a benefit that allows eligible veterans to receive covered care from providers in their neighborhood instead of a lengthy wait or long trip to be seen within a VHA facility, and other non-VHA care that veterans may receive.

The center box on the figure also presents *the Levels of Care Intervention* that reflects how SBDH and other risk factors can be addressed within the VHA as a large integrated delivery system. At the first level, SBDH factors' incorporation could help clinicians to better address socio-behavioral risk factors among patients within their patient panel. At the second level the VHA provider network could better design programs addressing “high-need, high-cost” patients. And at the third level VHA and other government agencies could use the risk factor information for those veteran communities, who are experiencing high levels of socio-behavioral risk and may require special outreach programs to address those in need [13].

The throughput part of the conceptual framework (bottom center box of Figure 1) is our summation of key aspects of the optimal *Patient & Population Health Analytics* process that the VHA providers could undertake using digitally collected SBDH information. The left-most part of this box outlines three *Analytics Supported Tasks*. These include: (1) *Patient Care & Management*, where SBDH factors could be used to help augment clinical interventions and clinically-oriented high-risk case identification as well as predictive models. For instance, SBDH risk factors for individual veterans and those associated with the veteran's neighborhood could be added into the VHA's medically oriented Care Assessment Need (CAN) score [22] which is now used to help estimate probability of admission at a specified time period; (2) *Risk Stratification*, where SBDH factors help adjust for potential differences in outcomes or costs [23]. For instance, the VHA could consider stratifying for different socioeconomic factors of veterans when assessing primary care quality metrics or resource expenditures; (3) *Community Assessment*, where SBDH information could play a key role in targeting communities or sub-populations with the highest risk and thus the best targets for special programmatic interventions. It is also possible that changes in the level of social and behavioral risk factors could serve as end-points and outcomes in their own right, helping to assess the impact of VHA population health programs.

The right part of the bottom population health analytics box acknowledges the three potential *SBDH Data Sources* obtained from each of the entities: The individual patient/consumer, the healthcare organization, and from sources associated with government or private entities that comprise the infrastructure of a geographically defined community. It should be noted that each of the data sources may be used to accomplish any of the three analytics supported tasks noted in the adjacent box to the left, or they may be used to target interventions at any of the three levels of care intervention noted in the adjacent box above. For example, consumer surveys on social and behavioral risk could be used at the patient level or aggregated across a PCMH or neighborhood (as is the case of the U.S. Census Bureau's American Community Survey or ACS) [24]. Another example would be risk factors derived from a provider's electronic health record (EHR). These data could be used to identify social and behavioral risk factors that are aggregated at the patient, clinic, or neighborhood level. Neighborhood characteristics (e.g., poor housing stock) can likewise be linked to patients or organization, in addition to neighborhoods from which the data are collected [16].

## 4. Individual and population level social and behavioral risk measures and assessment of available data

A key component of the proposed framework is the set of social, behavioral, and clinical risk factors in the leftmost box. To address these factors we recommended a comprehensive set of individual and population-level social and behavioral risk measures associated with individual veterans and the communities where they reside. We recommended SBDH risk factors for consideration relevant for integration into the analytic platforms of VHA's PCMH program or other VHA's patient-and population-oriented initiatives. Table 1 presents the SBDH domains and sub-domains, using National Academy of Medicine recommendations [18].

The recommended SBDH domains suggested in Table 1 include those related to service provision, geographic and individual linked health determinants, and medical, social, and behavioral outcomes. They address major SBDH impacting health such as sociodemographic, psychosocial, and behavioral characteristics [10], different aspects of housing [1],[20],[25]–[35] such as homelessness, housing insecurity, and characteristics of housing. Physical and built environment issues affecting walkability and access to healthcare, such as geographic characteristics of the living space, access to different modes of transportation, and social characteristics of the neighborhood such as safety are addressed as well [36]–[40]. It is also important to assess the role of psychosocial factors on accessibility of healthcare services. While it is rather widely observed that people with limited socioeconomic resources have limited access to healthcare, some vulnerable populations are less likely to recognize the importance of regular usage of primary healthcare, due to a wide range of psychosocial factors. Issues related to diet and food systems, namely access to healthy food options, public assistance food access, and healthy food habits [1],[18]–[45]; and socioeconomic issues such as income, education, employment, and neighborhood socioeconomic status are also presented [1],[20].

**Table 1.** Domains of social and behavioral determinants of health for consideration in VHA primary care clinics*.

SBDH Domains	SBDH Sub-domains		
Sociodemographic	Sexual Orientation		
	Race/Ethnicity		
	Country of Origin		
	Education		
	Employment		
	Financial Resource Strain	Food Insecurity	Public Assistance Food Access
			Individual's Food Intake
		Housing Insecurity**	
Psychological	Health Literacy		
	Stress		
	Negative Mood & Affect	Depression	
		Anxiety	
	Psychological Assets	Self-efficacy, Conscientiousness, Patient engagement/Activation, Optimism	
Behavioral	Dietary Patterns	Healthy Food Habits	
	Physical Activity		
	Tobacco Use and Exposure		
	Alcohol Use		
Social Relationship	Social Connection and Social Isolation		
	Violence Exposure		
Neighborhood Compositional Characteristics	Natural Environment	Air Quality	
		Water Quality	
		Childhood Lead Poisoning Levels	
	Physical/Built Environment	Housing	Housing Characteristics
			Housing Insecurity**
			Homelessness
		Walkability and Access	Geographic Characteristics of Living Space
			Mode of transportation
			Walkability Index
			Street Connectivity
			Access to Healthy Food Options
			Access to Healthcare Facility
	Socio-economic	Social Deprivation	
		Social Characteristics of Neighborhood	Income
			Education
			Employment
			Neighborhood Socioeconomic Index
		Economic Distress	
		Healthcare Access	
	Race/Ethnicity	Neighborhood-level Racial Residential Segregation	

Note: * The conceptual framework is presented in Figure 1. This table expands on categories of social and behavioral determinants of health (red circle in the figure) using recommendations from National Academy of Medicine.

** There are overlaps among different domains of SBDH. For instance, housing insecurity would be included in two domains.

SBDH: Social and Behavioral Determinants of Health; VHA: Veterans Health Administration.

In an appendix table, we provide a set of measures for each subdomain of SBDH. We considered these measures an initial set for the VHA's PCMH program to complement current SBDH measures available to them. The individual level and neighborhood linked SBDH were selected from a much larger set of candidate domains and measures based on the results of the literature review [10],[26],[46]–[54]. We assessed evidence on the relationship of SBDH with meaningful health outcomes or other endpoints, the availability of reliable measures, and the existence of digital data sources to allow measurement in the majority of locales across the U.S. We then narrowed down the list of measures to those presented. Table 2 presents our selection criteria for SBDH measures [55]. While this list includes a wide range of SDBH it is not an exhaustive one. In the past few years a large number of studies have assessed the impact of different SBDH on health outcomes and healthcare utilization.

As noted, we also provided an assessment of potential available data sources for each sub-category of social and behavioral factors not currently available in the VHA's electronic databases. The appended table of recommended SBDH measures and URL links to the current database or other resources can be used to calculate the SBDH relevant metrics on a population level and in most neighborhoods. The table also includes recommendations for inclusion of SBDH on a patient level in the VHA's electronic databases.

**Table 2.** Selection criteria for social and behavioral determinants measures.

Categories	Characteristics
Patient vs. Population/Neighborhood Focused	Relevant to patient or neighborhood level interventions
	Health System Interventions (e.g., VHA PCMH)
	Bringing population issues into clinical services (e.g. PCP, care manager, or outreach nurse)
Importance/Applicability	Patient or Population-based performance measures
	Factors that are important to take into account for patient care and population health interventions
Development of a Balanced Score Card for Patient Care and Population Health	Measures not related to clinical care (i.e., behavioral and social)
	Focusing on population facets of clinical care (i.e., the full denominator of those in need not just those getting care)
	Focusing on interplay between patient care and population health interventions
	A type of structure oriented QI measure that will serve as a motivator to help build new infrastructure for data collection for patient care and population health (e.g., a metric assessing the collection of SES data in EHRs)
	Tools that will support not just the current interventions, but also future innovations
	Relevant to small areas, i.e. when defining communities, we can go beyond just county or zip code
	Range of temporality, some measures address short term outcomes, others address longer-term outcomes
Overall Practicality and Strategic Value	Measurement areas previously addressed but where further work is needed
	Could be accomplished with limited resources (e.g., not a new major neighborhood survey)
	Fills a gap on the comprehensive framework we developed
Data Feasibility/Supports and Expands digital infrastructure	Data currently are available digitally or could be available in next few years
	Capitalizes and expands on new data assets (e.g., EHR)
Scientific Evidence/Measures Attributes	Some evidence that measures matter for health and welfare
	Ideally some preliminary measurement work exists
	Some previous validation of accuracy/feasibility desirable
	Some previous measure standards/certification desirable

Note: EHR: Electronic Health Record, PCMH: Patient-Centered Medical Home, PCP: Primary Care Provider, QI: Quality Improvement, SES: Socio-economic Status, VHA: Veterans Health Administration.

It is important to note that while some U.S. EHR vendors have started adding specific fields for collecting SBDH, no universally accepted and standardized format currently exists for documenting SBDH in EHRs [18],[33],[56]–[59]. Major efforts are underway to increase the standardized vocabulary and content of EHR data across the U.S. [60],[61], which would eventually impact the quality and coverage of SBDH documentation in EHRs. For example, Centers for Medicare and Medicaid Services (CMS) required the collection of demographic information including race, ethnicity, and preferred language, and smoking status as the core measures in stage 1 of the Meaningful Use (MU) program [62]. Also, CMS now requires that all in-scope clinicians apply standardized processes and definitions within their certified EHR to screen for and document SBDH concerning food security, employment, and housing [63]. Such initiatives are fiscally backed by Medicare and might offer a successful framework for the collection of consistent SBDH data across EHRs. While these initiatives do not directly impact VHA, the standardized approach to SBDH documentation in EHRs will eventually affect all EHR systems across the spectrum of healthcare organizations.

## 5. Current challenges and future steps for integration of SBDH into healthcare analytics

A number of challenges should be addressed in order to systematically identify and respond to the socio-behavioral factors that have the highest impact on health outcomes and healthcare cost. VHA, similar to most of the care delivery systems in the country and other healthcare organizations, lacks analytic frameworks to incorporate information about patients' social and behavioral risk and neighborhood characteristics into clinical or organizational decision-making. They also lack access to SBDH data sources in the electronic databases for each sub-domain of social and behavioral factors on a patient level, for subpopulations, or for a neighborhood at-large [11]. Most healthcare organizations often use administrative claims and structured EHRs for assessment of SBDH, which lacks in-depth assessment of important social and behavioral factors affecting health, such as housing issues and food insecurity. The limited access to data on social and behavioral factors through EHRs' unstructured free-text, which requires time-consuming and subjective methods such as chart review for identification of patients with high social and behavioral risk, is not a feasible approach for screening a large population of patients [25],[46],[48],[63]. Application of supplementary social and behavioral data on a population level from national surveys such as ACS by the U.S. Census Bureau [24] or limited clinic-based surveys are not easily available across different populations and healthcare organizations [25],[46].

The evidence-based framework and the initial set of individual and neighborhood level social and behavioral risk measures that we proposed here would help to develop analytic platforms for systematic identification of SBDH and their integration in clinical decision supports and different levels of care within VHA and other large integrated delivery systems as well as smaller, less-integrated systems. Increased integration of such information into patient care could allow the physicians and other clinicians to tailor personalized care and modulate care coordination efforts as needed [11],[13]. Examples here include a clinician's knowledge of a diabetic patient's social and behavioral risk factors such as living in a food desert or having food insecurity would help them to provide personalized recommendations regarding their weight management. It would also help the physician in connecting the patient to potential social services such as Supplemental Nutrition Assistance Program (SNAP) and Special Supplemental Nutrition Program for Women, Infants, and Children (WIC) programs [64],[13].

Our framework also presented the key aspects of the optimal analytics process that VHA's PCMH program or other integrated providers would undertake to perform analytics-supported tasks such as patient care and management, risk stratification, and community assessment, using digitally collected SBDH information [55]. The optimal analytics process could facilitate the community engagement to strengthen links between clinics and community resources [65].

The three proposed potential SBDH data sources obtained from the individual patient/consumer, the healthcare organizations, and from a geographically defined neighborhood could guide VHA and other healthcare organizations to link data within their organization and to supplement social and behavioral data on a population level. This linkage of different data sources would enable them to address SBDH in their decision-making and program development while investing in standardized processes to document SBDH and collect high-quality patient-level data for SBDH assessment.

Linking population-level data such as those obtained by the U.S. Census Bureau [24] to data collected in EHRs and other clinical and administrative patient-level information systems helps to identify patients at risk of poor health outcomes and high social needs. Assessing the geographic variations in health outcomes and healthcare utilization such as hospitalization and emergency department visits based on demographic, clinical, and socioeconomic factors could also signal differential access to care and disparities in quality of care [47]. The application of placed-based data to assess disparities at the geographic level or other population approaches in healthcare is a powerful tool for case management purposes of underserved populations. Using data available in small geographic areas such as a census tract or block group can help in identifying those neighborhoods with low socioeconomic characteristics suffering from different social and health issues which require intervention; and, provide evidence to support healthcare policies and resource allocation to patients and communities in need.

Using population level SBDH data would be the best option when the intervention is designed at a neighborhood level (e.g., eliminating food desserts) or when there is lack of individual level data for a specific issue (e.g., how far a patient lives from a food outlet). However, in other instances, when interventions are designed at the individual-level and data is available using individual-level data is more informative. For instance, the economic status of an individual patient and the economic profile of the neighborhood in which she lives are both important, but the former has a more important impact on healthcare utilization and health outcomes.

Our proposed framework and recommended SBDH metrics can provide a road map for VHA and other primary care-centric healthcare organizations wishing to use the analytic tools to better understand how SBDH affect health outcomes. It would enable them to target their limited care management resources to “high-need, high-cost” patients and would provide the platform for health systems and public health agencies to collaborate in improving the health of the population [55],[66]. Future research should assess the impact of such systematic approach to identification of SBDH at different levels of care within a healthcare system on health outcomes and utilization of healthcare services.
