# Supplementary material for: Integrating social and behavioral determinants of health into patient care and population health at Veterans Health Administration: a conceptual framework and an assessment of available individual and population level data sources and evidence-based measurements
Source: AIMS Public Health. 2019 Jul 3;6(3):209–24. doi: 10.3934/publichealth.2019.3.209 (PMC6779595; doi:10.3934/publichealth.2019.3.209)
Supplement: Supplementary file 1 [file publichealth-06-03-209-s001.pdf]

Public Health

AIMS Public Health, 6(3): 209–224.
DOI: 10.3934/publichealth.2019.3.209
Received: 15 May 2019
Accepted: 24 June 2019
Published: 03 July 2019

http://www.aimspress.com/journal/aimsph

Commentary

Integrating social and behavioral determinants of health into patient care and population health at Veterans Health Administration: a conceptual framework and an assessment of available individual and population level data sources and evidence-based measurements

Elham Hatef[1,*], Zachary Predmore[1], Elyse C. Lasser[1], Hadi Kharrazi[1], Karin Nelson[2,3], Idamay Curtis[2], Stephan Fihn[2,3] and Jonathan P. Weiner[1]

[1] Center for Population Health IT, Department of Health Policy and Management, Johns Hopkins Bloombe[illegible] Health, Baltimore, MD, USA
[2] Veterans Affairs Puget Sound Health Care System, Seattle, WA, USA
[3] Department of Medicine, University of Washington School of Medicine, Seattle, WA, USA

* **Correspondence:** Email: ehatef1@jhu.edu; Fax: +4432872284.

Appendix Table

Appendix Table. Recommended measures and data sources to address social and behavioral determinants of health in VHA primary care clinics.

SBDH Domains	SBDH Sub-domains	Recommended Measures	Data Source & Data Level
Sociodemographic	Sexual Orientation	Sexual orientation—identified by an individual<br>Gender identity—recorded on individual's birth certificate or the gender an individual identifies with<br>Sexual behavior—preference for sexual relationship	EHR[1,2,3] Individual Level Data
	Race/Ethnicity	American Indian or Alaska Native, Asian, Black or African American, Native Hawaiian or Other Pacific Islander, White, and other. Ethnicity includes two categories: Hispanic or Latino, and Not Hispanic or Latino	EHR[2,3,4,5,6] Individual Level Data
	Country of Origin	U.S. born or non-U.S. born and spoken language or preferred language for healthcare	EHR[2,3,4,5,6] Individual Level Data
	Education	Number of years of time formally spent in school and the highest degree earned	EHR[2,6] Individual Level Data
	Employment	Formal paid labor market (including self-employment) and type of employment and the conditions that this implies, including exposure to health risks and hazards in the workplace, income, stress, and provision of health care insurance	EHR[2,6] Individual Level Data

Continued on next page

SBDH Domains	SBDH Sub-domains			Recommended Measures	Data Source & Data Level
Sociodemographic	Financial Resource Strain	Food Insecurity	Public Assistance Food Access	Those using SNAP program for public assistance food access	EHR Individual Level Data[7] & ACS[8], Food Access Research Atlas[9] Population Level Data
			Individual's Food Intake	NHANES III[10]—Which of the following describes the amount of food patient's household has to eat: Enough to eat, Sometimes not enough to eat, Often not enough to eat	EHR[2] Individual Level Data
		Housing Insecurity*		Multiple moves, crowding, and foreclosure	EHR[6,7] Individual Level Data
Psychological	Health Literacy[2]			The degree to which individuals have the capacity to obtain, process, and understand basic health information and services needed to make appropriate health decisions	EHR[2,3,6] Individual Level Data
	Stress			Stress means a situation in which a person feels tense, restless, nervous or anxious or is unable to sleep at night because his/her mind is troubled all the time. Does patient feel these kinds of stress these days? A 5-point Likert scale response varying from 1 "not at all" to 5 "very much"	EHR[2,3,6] Individual Level Data

Continued on next page

SBDH Domains	SBDH Sub-domains		Recommended Measures	Data Source & Data Level
Psychological	Negative Mood & Affect	Depression/Anxiety	PHQ-2 Questionnaire[3,11]—Over the past 2 weeks, how often has the patient been bothered by any of the following problems:<br>1. Little interest or pleasure in doing things<br>Not at all, Several days, More than half the days, Nearly every day<br>2. Feeling down, depressed or hopeless<br>Not at all, Several days, More than half the days, Nearly every day	EHR[2,3,6] Individual Level Data
	Psychological Assets	Conscientiousness, Patient engagement/Activation, Optimism, and Self-efficacy[2]	NIH toolbox self-efficacy survey; the extent to which an individual believes in their ability to change, contains 10 questions measured on a scale of 0-100 in intervals of 10 (0 = minimum 100 = max self-awareness/ability to change)	EHR[2] Individual Level Data & NIH Toolbox[12] Population Level Data
Behavioral	Dietary Patterns	Healthy Food Habits	Fruits and vegetables spending; average annual fruit and vegetable spending per household and average annual spending on food at home by type of food (information on different eating habits of communities in terms of consumption of food, beverages, organic vs non-organic)	National Household Food, Acquisition and Purchase Survey (FoodAPS)[13], ESRI Consumer Spending Database, American Time Use Survey Eating and Health[14] Population Level Data

Continued on next page

SBDH Domains	SBDH Sub-domains	Recommended Measures	Data Source & Data Level
Behavioral	Physical Activity	On average, how many days per week does the patient engage in moderate to strenuous exercise (like walking fast, running, jogging, dancing, swimming, biking, or other activities that cause a light or heavy sweat)? On average, how many minutes does the patient engage in exercise at this level?	EHR[2] Individual Level Data
	Tobacco Use and Exposure	NHIS[3,15]—First or second hand smoking, current/past smoker duration, and quantity	EHR[2,3] Individual Level Data
	Alcohol Use	AUDIT-C[16]—How often does the patient have a drink containing alcohol? Never, Monthly or less, 2–4 times a month, 2–3 times a week, 4 or more times a week How many standard drinks containing alcohol does the patient have on a typical day? 1 or 2, 3 or 4, 5 or 6, 7 to 9, 10 or more How often does the patient have six or more drinks on one occasion? Never, Less than monthly, Monthly, Weekly, Daily or almost daily	EHR[2,3] Individual Level Data
Social Relationship	Social Connections and Social Isolation[17,18,19,20,21]	NHANES III	EHR[2,3,6] Individual Level Data
		The proportion not currently married (% unmarried ages ≥ 15: overall population and for race and ethnicity) and also living arrangement (the percentage of people living alone, or living with an unmarried partner)	ACS[8] Population Level Data–Population Level Data

Continued on next page

SBDH Domains	SBDH Sub-domains		Recommended Measures	Data Source & Data Level
Social Relationship	Social Connections and Social Isolation[17,18,19,20,21]		Estimates of how, where, and with whom Americans spend their time, and the full range of nonmarket activities, from childcare to volunteering	The American Time Use Survey[14] Population Level Data
	Violence Exposure		Behavior that threatens, attempts or causes physical harm—HARK self-reported instrument;[22] Within the last year, has the patient been humiliated or emotionally abused in other ways by the partner or ex-partner? Yes No Within the last year, has the patient been afraid of the partner or ex-partner? Yes No Within the last year, has the patient been raped or forced to have any kind of sexual activity by the partner or ex-partner? Yes No Within the last year, has the patient been kicked, hit, slapped, or otherwise physically hurt by the partner or ex-partner? Yes No	EHR[2,3] Individual Level Data
Neighborhood Compositional Characteristics	Natural Environment	Air Quality[23]	Number of person-days with maximum 8-h average ozone concentration over the national ambient air quality standard (monitored and modeled data) Number of person-days with PM2.5 over the national ambient air quality standard (monitored and modeled data)	CDC Environmental Public Health Tracking Network[24] Population Level Data

Continued on next page

SBDH Domains	SBDH Sub-domains		Recommended Measures		Data Source & Data Level
Neighborhood Compositional Characteristics	Natural Environment	Water Quality	Percent of population potentially exposed to water exceeding a violation limit during the past year		States' Safe Drinking Water Act Databases—Safe Drinking Water Information System[25] Population Level Data
		Childhood Lead Poisoning Levels[26]	State surveillance data for children age < 72 months who were tested for lead at least once since January 1, 1997		CDC National Surveillance Data[27] Population Level Data
	Physical/Built Environment	Housing[17,18,23,28,20,30,31,32,33,34,35,36,37,38]	Housing Characteristics	Number of units in structure, year structure built, number of rooms, average household size, house heating fuel, plumbing, kitchen facilities, available telephone service, occupants per room, value of the house	ACS[8] Population Level Data
			Housing Insecurity*	Geographical mobility in the past year for current residence--state, county and place level, residential stability, housing unit vacancy rate, Homeownership Rate	ACS[8] and The American Housing Survey[39] Population Level Data

Continued on next page

SBDH Domains	SBDH Sub-domains		Recommended Measures		Data Source & Data Level
Neighborhood Compositional Characteristics	Physical/Built Environment	Housing[17,18,23,28,20,30,31,32,33,34,35,36,37,38]	Homelessness	Annual PiT estimates of homelessness in different population in a single night	Department of Housing and Urban Development[40] Population Level Data
		Walkability and Access[41,42,43,44,45]	Geographic Characteristics of Living Space	Living in rural vs urban areas	2000 Census Population Level Data
			Mode of transportation	Mobility network (access to public transport and private cars)	ACS[8] and Transit data[46] Population Level Data
			Walkability Index	Composite measure: density-residential and employment (total activity units per acre of land); diversity-land use and destinations (range of land uses by census tract); design-built environment and safety feature (Number of street crossings by census tract); distance- transit accessibility (aggregate frequency of transit service per square mile)	ACS[8] and Transit data[46] Population Level Data
			Street Connectivity	Intersection density (number of three-way or greater intersections per unit area)	ACS[8] Population Level Data

Continued on next page

SBDH Domains	SBDH Sub-domains		Recommended Measures		Data Source & Data Level
Neighborhood Compositional Characteristics	Physical/Built Environment	Walkability and Access[41,42,43,44,45]	Access to Healthy Food Options	Food insecurity/food desert	Food Access Research Atlas[9] Population Level Data
				Food retailers: count of businesses by type such as grocery stores, meat and fish markets, fruit and vegetable markets, candy nut and confectionary stores, retail bakeries, misc. food stores, distances between person's homes to the nearest grocery store, convenience store, fast food restaurant, and park (shortest network path for walking, and fastest network path for driving)	referenceUSA,[47] ESRI Business Analyst Data,[48] U.S. Census Bureau; County Business Patterns[49] Population Level Data
				Number of fast food restaurants and liquor stores per 100,000 population	U.S. Census Bureau; County Business Patterns[49] Population Level Data

Continued on next page

SBDH Domains	SBDH Sub-domains		Recommended Measures		Data Source & Data Level
Neighborhood Compositional Characteristics	Physical/Built Environment	Walkability and Access[41,42,43,44,45]	Access to Healthcare Facility	Distance from healthcare facility (address/zip code of healthcare facilities)	American Hospital Association Data[50] Population Level Data
	Socio-economic	Social Deprivation Index[19]	Composite measure: percent living in poverty; black; less than 12 years of schooling; single parent households; single occupant households; percent living in overcrowded conditions (more persons in a dwelling unit than number of rooms); percent of households without a car; percent with high needs measure (the percent of the population under the age of 5 and female between the ages of 15 and 44); and percent of 18- to 64-year old that are unemployed		ACS[8] Population Level Data
		Social Characteristics of Neighborhood	Income[23,51]	Median household income, income self-reported into categories (<$15k; $15k-29,999; 30k-44,999; 45k-59,999; 60k-89,999, >/=90k)	ACS[8] Population Level Data
			Education[52]	School enrollment by detailed level of school for the population 3 years and over, educational attainment for the population 25 Years and over	ACS[8] Population Level Data
			Employment[30]	Detailed occupation for the full-time, year-round civilian employed population 16 years and over	ACS[8] Population Level Data

Continued on next page

SBDH Domains	SBDH Sub-domains		Recommended Measures		Data Source & Data Level
Neighborhood Compositional Characteristics	Socio-economic	Social Characteristics of Neighborhood	Neighborhood Socioeconomic Index[23,53]	Composite Measure: percentage of adults age 25 years or older with less than a high school education; percentage of men who are unemployed; percentage of households with income below the poverty level; percentage of households receiving public assistance; percentage of households with children in which the head of household is female; and median household income	ACS[8] Population Level Data
		Economic Distress	Denial of the loan and the reason for denial, loan delinquency defined as delinquent loans and leases are those past due thirty days or more and still accruing interest as well as those in nonaccrual status		Federal Financial Institution Examination Council[54], The Federal Deposit Insurance Corporation[55] Population Level Data
		Healthcare Access	Health insurance, Medicare, VHA healthcare coverage status by age and sex		ACS[8] Population Level Data
	Race/Ethnicity	Neighborhood-level Racial Residential Segregation[23,56,57,]	Dissimilarity index (most common measure), isolation index, index of concentration at extremes (ICE), Atkinson Index, entropy, Gini coefficient, the Getis-Ord Gi* statistic		Census 2010 Population Level Data

Note: * There are overlaps among different domains of SBDH. For instance, housing insecurity would be included in two different domains.

ACS: American Community Survey, AUDIT-C: Alcohol Use Disorders Identification Test (AUDIT), CDC: Center for Disease Control and Prevention, EHR: Electronic Health Record, ESRI: Environmental Systems Research Institute, HARK: Humiliation, Afraid, Rape, Kick. A 4 item questionnaire to identify intimate partner violence, NHANES III: the third National Health and Nutrition Examination Survey, NIH: National Institute of Health, NIHS: National Health Interview Survey, PHQ-2: Patient Health Questionnaire-2, PiT: Point-in-Time, PM2.5: atmospheric particulate matter (PM) that have a diameter of less than 2.5 micrometers, SBDH: Social and Behavioral Determinants of Health, SNAP: Supplemental Nutrition Assistance Program, VHA: Veterans Health Administration.

References for the Appendix Table

1. Institute of Medicine (2013) *Collecting Sexual Orientation and Gender Identity Data in Electronic Health Records: Workshop Summary,* Washington, DC: The National Academies Press.
2. Board on Population Health and Public Health Practice, Institute of Medicine of The National Academies (2014) *Capturing social and behavioral domains and measures in electronic health records: Phase 2.* Available from: https://www.nap.edu/resource/18951/EHRreportbrief.pdf.
3. Gold R, Cottrell E, Bunce A, et al. (2017) Developing electronic health record (EHR) strategies related to health center patients' social determinants of health. *J Am Board Fam Med* 30: 428–447.
4. Centers for Medicare and Medicaid Services. Meaningful Use Stage 1 Requirements Overview. Available from: https://www.cms.gov/Regulations-and-Guidance/Legislation/EHRIncentivePrograms/downloads/mu_stage1_reqoverview.pdf.
5. Institute of Medicine (2009) *Race, Ethnicity, and Language Data: Standardization for Health Care Quality Improvement,* Washington, DC: The National Academies Press.
6. National Association of Community Health Centers. Protocol for Responding to and Assessing Patients' Assets, Risk, and Expernces. Available from: http://www.nachc.org/research-and-data/prapare/.
7. Centers for Medicare and Medicaid Services. Updated 2018 CMS QRDA III Implementation Guide for Eligible Clinicians and Eligible Professionals. Available from: https://ecqi.healthit.gov/ecqms/ecqm-news/now-available-updated-2018-cms-qrda-iii-implementation-guide-eligible-clinicians-0.
8. The United States Census Bureau. American community survey (ACS). Available from: https://Www.census.gov/programs-surveys/acs/.
9. United States Department of Agriculture (2017) Economic Research Service. Food access research atlas. Available from: https://www.ers.usda.gov/data-products/food-access-research-atlas/.
10 Alaimo K, Briefel RR, Frongillo EA, et al. (1998) Food insufficiency exists in the United States: Results from the third National Health and Nutrition Examination Survey (NHANES III). *Am J of Public Health* 88: 419–426.
11. Kroenke K, Spitzer RL, Williams JBW (2003) The Patient Health Questionnaire-2: Validity of a two-item depression screener. *Medical Care* 41: 1284–1292.
12. Health Measures (2018) Transforming how health is measured. Available from: http://www.healthmeasures.net/explore-measurement-systems/nih-toolbox.
13. United States Department of Agriculture (2017) Economic Research Service. FoodAPS national household food acquisition and purchase survey. Available from: https://www.ers.usda.gov/data-products/foodaps-national-household-food-acquisition-and-purchase-survey/.
14. Bureau of Labor Statistics (2016) American Time Use Survey. Available from: https://www.bls.gov/tus/.
15. Center for Disease Control and Prevention (2014) Current cigarette smoking among adults—United States, 2005–2012. *Morbidity and Mortality Weekly Report* 63: 29–46.

16. Bush K, Kivlahan DR, McDonell MD, et al. (1998) The AUDIT alcohol consumption questions (AUDIT-C): An effective brief screening test for problem drinking. Ambulatory Care Quality Improvement Project (ACQUIP). Alcohol use disorders identification test. *Arch Intern Med* 158: 1789–1795.
17. National Academies of Sciences, Engineering, and Medicine (2017) *Accounting for social risk factors in Medicare payment: Identifying social risk factors,* Washington, DC: The National Academies Press.
18. Navathe AS, Zhong F, Lei VJ, et al. (2018) Hospital readmission and social risk factors identified from physician notes. *Health Serv Res* 53: 1110–1138.
19. Butler DC, Petterson S, Phillips RL, et al. (2013) Measures of social deprivation that predict health care access and need within a rational area of primary care service delivery. *Health Serv Res* 48: 539–559.
20. Gant Z, Lomotey M, Hall HI, et al. (2012) A county-level examination of the relationship between HIV and social determinants of health: 40 states, 2006–2008. *Open AIDS J* 6: 1–7.
21. Berkman LF, Syme SL (1979) Social networks, host resistance, and mortality: A nine-year follow-up study of alameda county residents. *Am J Epidemiol* 109: 186–204.
22. Sohal H, Eldridge S, Feder G (2007) The sensitivity and specificity of four questions (HARK) to identify intimate partner violence: A diagnostic accuracy study in general practice. *BMC Fam Pract* 8: 49–58.
23. Bazemore AW, Cottrell EK, Gold R, et al. (2016) Community vital signs: Incorporating geocoded social determinants into electronic records to promote patient and population health. *J Am Med Inform Assoc* 23: 407412.
24. Center for Disease Control and Prevention (2017) National environmental public health tracking network. Available from: https://ephtracking.cdc.gov/showHome.action.
25. United States Environmental Protection Agency (2016) The safe drinking water information system (SDWIS). Available from: https://www.epa.gov/enviro/data-downloads.
26. Center for Disease Control and Prevention (2017) National center for environmental health (NCEH). Available from: https://www.cdc.gov/nceh/.
27. Center for Disease Control and Prevention (2016) National Surveillance Data. Available from: https://www.cdc.gov/nceh/lead/data/national.htm.
28. Nagasako EM, Reidhead M, Waterman B, et al. (2014) Adding socioeconomic data to hospital readmissions calculations may produce more useful results. *Health Aff (Millwood)* 33: 786–791.
29. The Center for Health Care Strategies (2016) Measuring social determinants of health among low-income populations: Early insights from state initiatives. Available from: http://www.chcs.org/resource/measuring-social-determinants-health-among-low-income-populations-early-insights-state-initiatives/.
30. Remington PL, Catlin BB, Gennuso KP (2015) The county health rankings: Rationale and methods. *Popul Health Metr* 13: 11.
31. Taylor LA, Tan AX, Coyle CE, et al. (2016) Leveraging the social determinants of health: What works? *PLoS One* 11: e0160217.

32. Buck DS, Brown CA, Mortensen K, et al. (2012) Comparing homeless and domiciled patients' utilization of the Harris county, Texas public hospital system. *J Health Care Poor Underserved* 23: 1660–1670.
33. Brown RT, Miao Y, Mitchell SL, et al. (2015) Health outcomes of obtaining housing among older homeless adults. *Am J Public Health* 105: 1482–1488.
34. Juhn YJ, Beebe TJ, Finnie DM, et al. (2011) Development and initial testing of a new socioeconomic status measure based on housing data. *J Urban Health* 88: 933–944.
35. Burgard SA, Seefeldt KS, Zelner S (2012) Housing instability and health: Findings from the Michigan recession and recovery study. *Soc Sci Med* 75: 2215–2224.
36. Rohe WM, Stewart LS (1996) Homeownership and neighborhood stability. *Housing Policy Debate* 7: 37–81.
37. Executive Office of Health and Human Services (2016) Mass health risk adjustment model social determinants of health. Available from: http://www.mass.gov/eohhs/docs/eohhs/healthcare-reform/masshealth-innovations/1610-risk-adjustment-open-public-meeting.pdf.
38. Ash AS, Mick E (2016) UMass risk adjustment project for mass health payment and care delivery reform: Describing the 2017 payment model. Available from: http://www.mass.gov/eohhs/docs/eohhs/healthcare-reform/masshealth-innovations/1610-umass-modeling-sdh-summary-report.pdf.
39. The U.S. Census Bureau. American Housing Survey (AHS) (2017) Available from: https://www.census.gov/programs-surveys/ahs.html.
40. U.S. Department of Housing and Urban Development (2017) The homelessness data exchange. Available from: http://www.hudhdx.info.
41. Virginia Department of Health (2012) Virginia health opportunity index (HOI). Available from: http://www.vdh.virginia.gov/health-equity/virginia-health-opportunity-index-hoi/.
42. Glazier RH, Creatore MI, Weyman JT, et al. (2014) Density, destinations or both? A comparison of measures of walkability in relation to transportation behaviors, obesity and diabetes in Toronto, Canada. *PloS one* 9: e85295.
43. Leonardi C, Simonsen NR, Yu Q, et al. (2017) Street connectivity and obesity risk: Evidence from electronic health records. *Am J Prev Med* 52: S40–S47.
44. Rohrer J, Pierce JR, Denison A (2004) Walkability and self-rated health in primary care patients. *BMC Fam Pract* 5: 29.
45. Zhu X, Lee C (2008) Walkability and safety around elementary schools economic and ethnic disparities. *Am J Prev Med* 34: 282–290.
46. The Center for Neighborhood Technology. AllTransitTM data. Available from: http://alltransit.cnt.org/faq/.
47. Reference USA (2017) Available from: http://resource.referenceusa.com.
48. ESRI Business Analyst (2017) ArcGIS business analyst. Available from: http://www.esri.com/software/businessanalyst/data-and-reports.
49. U.S. Census Bureau (2017) County business patterns. Available from: https://www.census.gov/programs-surveys/cbp.html.

50. American Hospital Association (2017) AHA data products. Available from: http://www.aha.org/research/rc/stat-studies/data-and-directories.shtml.
51. Auger KA, Kahn RS, Simmons JM, et al. (2017) Using address information to identify hardships reported by families of children hospitalized with asthma. *Acad Pediatr* 17: 79–87.
52. Barnett ML, Hsu J, McWilliams JM (2015) Patient characteristics and differences in hospital readmission rates. *JAMA Intern Med* 175: 1803–1812.
53. Nelson K, Schwartz G, Hernandez S, et al. (2017) The association between neighborhood environment and mortality: Results from a national study of veterans. *J Gen Intern Med* 32: 416–422.
54. Federal Financial Institution Examination Council. Central data repository's public data distribution web site. Available from: https://cdr.ffiec.gov/public/
55. The Federal Deposit Insurance Corporation (2017) Bank data and statistics. Available from: https://www.fdic.gov/bank/statistical/.
56. Moonesinghe R, Beckles GLA (2015) Measuring health disparities: A comparison of absolute and relative disparities. *Peer J* 3: e1438.
57. Kershaw KN, Robinson WR, Gordon-Larsen P, et al. (2017) Association of changes in neighborhood-level racial residential segregation with changes in blood pressure among black adults: The CARDIA study. *JAMA Intern Med* 177: 996–1002.

AIMS Press

© 2019 the Author(s), licensee AIMS Press. This is an open access article distributed under the terms of the Creative Commons Attribution License (http://creativecommons.org/licenses/by/4.0)
